# Characteristics of Plantar Loads in Maximum Forward Lunge Tasks in Badminton

**DOI:** 10.1371/journal.pone.0137558

**Published:** 2015-09-14

**Authors:** Xiaoyue Hu, Jing Xian Li, Youlian Hong, Lin Wang

**Affiliations:** 1 School of Kinesiology, Shanghai University of Sport, Shanghai, China; 2 School of Human Kinetics, University of Ottawa, Ottawa, Canada; West Virginia University, UNITED STATES

## Abstract

**Background:**

Badminton players often perform powerful and long-distance lunges during such competitive matches. The objective of this study is to compare the plantar loads of three one-step maximum forward lunges in badminton.

**Methods:**

Fifteen right-handed male badminton players participated in the study. Each participant performed five successful maximum lunges at three directions. For each direction, the participant wore three different shoe brands. Plantar loading, including peak pressure, maximum force, and contact area, was measured by using an insole pressure measurement system. Two-way ANOVA with repeated measures was employed to determine the effects of the different lunge directions and different shoes, as well as the interaction of these two variables, on the measurements.

**Results:**

The maximum force (MF) on the lateral midfoot was lower when performing left-forward lunges than when performing front-forward lunges (p = 0.006, 95% CI = −2.88 to −0.04%BW). The MF and peak pressures (PP) on the great toe region were lower for the front-forward lunge than for the right-forward lunge (MF, p = 0.047, 95% CI = −3.62 to −0.02%BW; PP, p = 0.048, 95% CI = −37.63 to −0.16 KPa) and left-forward lunge (MF, p = 0.015, 95% CI = −4.39 to −0.38%BW; PP, p = 0.008, 95% CI = −47.76 to −5.91 KPa).

**Conclusions:**

These findings indicate that compared with the front-forward lunge, left and right maximum forward lunges induce greater plantar loads on the great toe region of the dominant leg of badminton players. The differences in the plantar loads of the different lunge directions may be potential risks for injuries to the lower extremities of badminton players.

## Introduction

Badminton is a popular non-contact racket sport that requires athletes to perform jumps, lunges, quick directional changes, and rapid arm movements from a wide variety of postural positions [1]. Badminton can be considered an intermittent individual sport, characterized by combining moments of high intensity interspersed with short periods of low intensity or rest [2, 3]. In competitive badminton, footwork is the most fundamental skill. Excellent footwork allows players to reach the shuttlecock as quickly as possible with minimum effort and performance time [4]. Footwork enables players to move into the best position to execute shots while maintaining good balance and body control [5].

The lunge step accounts for 15% of all movements in a badminton singles match [6]. Players often perform powerful and long-distance lunges during such competitive matches. Sudden stop-and-go maneuvers, such as rapid and repetitive lunge steps that involve strenuous impact during heel contact, produce variable loads on the lower extremities and could thus induce injuries in this part of the body [7]. Therefore, investigating the kinetics and kinematics of lunge tasks may offer biomechanical information on enhancing athletic performance and provide coaches and players with a reference for the prevention of injuries during trainings and competitions. A few studies have investigated the biomechanical characteristics of the different forms of lunge footwork have been investigated [4, 6, 8, 9]. Kuntze et al. found that lunge patterns influence the kinetics of the lower extremities [6]. A recent study demonstrated that the left-forward lunge has higher vertical ground reaction force (vGRF), higher loading rate, and greater plantar pressure at the total foot and heel regions compared with the right-forward and left/right-backward lunges [4]. Fu et al. found that plantar pressure is concentrated in the heel and the lateral foot during forward lunges [8]. Among these biomechanical factors, repetitively high plantar pressure is a potential factor for sports-related injuries to the lower extremities [10]. During the impact phase of forward lunges, players exhibit vGRFs that are approximately 2.1 to 2.5 times their body weight (BW) [4, 6, 9].The feet experience a great amount of stress, which may lead to fatigue and painful conditions. Injuries to the lower extremities, such as pain in the Achilles tendon and knee joint, during badminton games account for 58% of all badminton-related injuries [11]. In addition, training surface, abnormal biomechanics, the performed task and footwear have also been reported to influence plantar pressure measurements [12, 13]. Therefore, identifying the impact forces and plantar pressure distribution characteristics during lunges at different directions may help reveal the risk factors related to sports injuries. To date, no quantitative information on the comparison of plantar load characteristics during maximum forward lunge tasks at different directions has been made available.

Considering the contention about the loading mechanics involved in different long-distance lunges, the aim of the study is to compare the insole load responses of three one-step maximum forward lunge tasks in badminton, namely, right-forward, left-forward, and front-forward lunges. Identifying these differences may effectively lead to the prevention of injuries during badminton trainings and competitions.

## Materials and Methods

### Participants

We based the sample size calculation on detecting a larger effect size among three tasks at an alpha level of 0.05. Based on this, 6 participants were required to show a large effect size (0.8) using a 2-tailed hypothesis. In the study, the participants of this study comprised 15 right-handed male badminton players with a shoe size of EUR 41. The players were all active participants in badminton singles competitions at the university level and had at least two years of competition experience in the sport. All the players reported that they had been free from neuromuscular, vestibular, and vision system injuries for at least six months before their participation in the study and that they were in generally good physical condition. Participants were excluded if they had any history of visual problems, any deformity in lower extremities or spine, previous history of surgery, neurological or systemic disorders. They were also excluded if they had taken any sedative drug or alcohol within the past 48 h. The study was approved by the Shanghai University of Sport. All the participants signed a written informed consent form before the test.

### Measurements

Primary outcomes measures of the study was plantar loads at the total foot and at each foot mask during maximal lunge tasks. Secondary outcome measures included body weight and height measurements.

Participants reported their age. Body mass and height were measured with minimal clothing and bare feet. Body height was measured to the nearest 0.5 cm using a fixed stadiometer. Body mass was measured to the nearest 0.1 kg using a standard scale. A trained investigator performed all the measurements.

Three pairs of badminton shoes were selected for the study. Each pair carried a different brand. Thus, three brands that are commonly used in competitions were considered. The three pairs of shoes were Li Ning (Model No. 2YMD649-1, Li-Ning Co., Ltd., China), Mizuno (Model No. 7KM-75562, Mizuno Co., Ltd., Japan) and Yonex (Model No. SHB-91MX, Yonex Co., Ltd., Japan). The shoes were coded as L, M, and Y respectively. In the study, the participants blinded to type of shoes. Symbols of the shoes were removed or shadowed.

**Fig 1:**
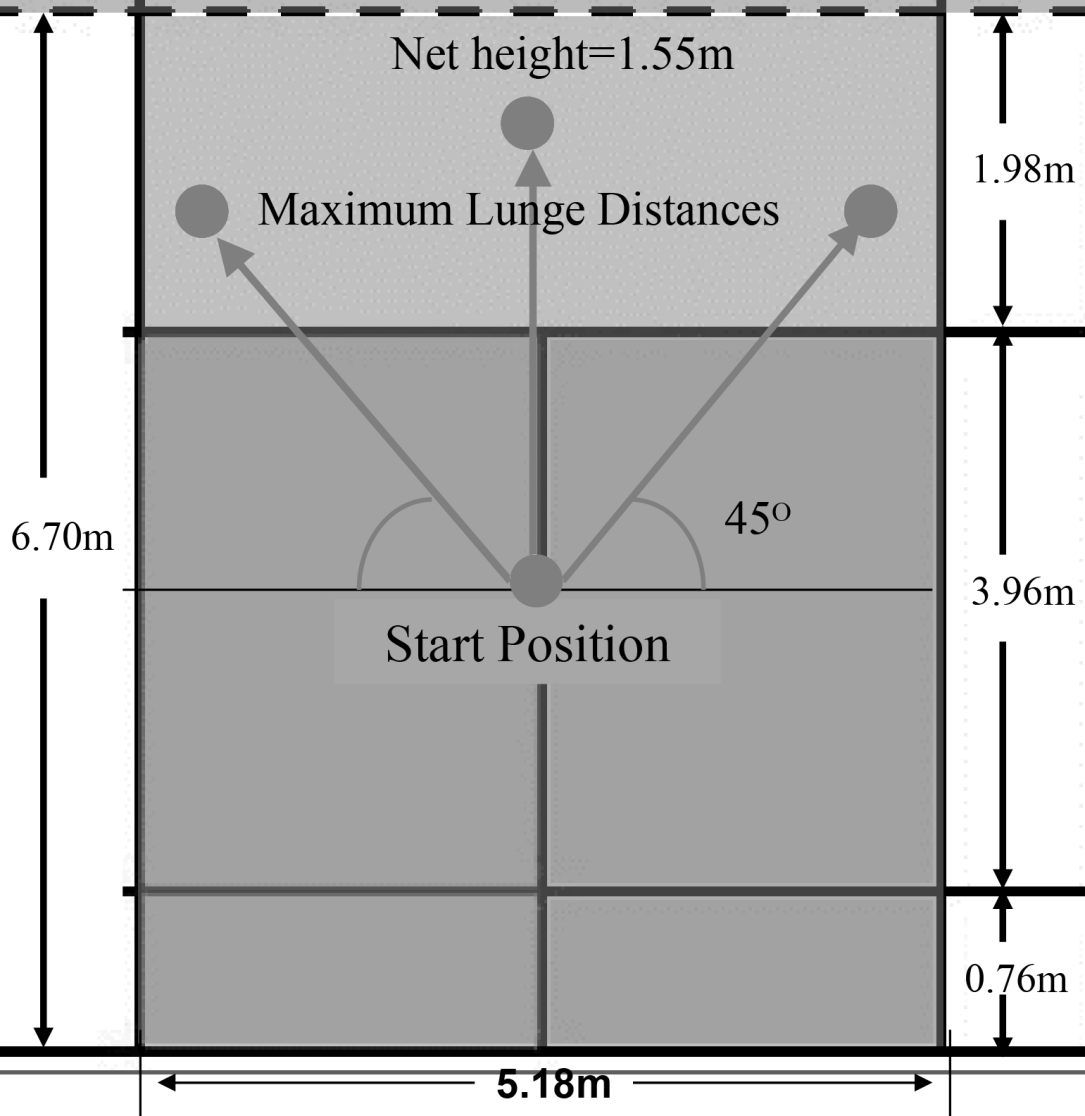
Experimental setup.Foot loading was measured by using an insole pressure measurement system (Pedar-X system, Novel, Munich, Germany). Each insole contained 99 force sensors with a spatial resolution of approximately 10 mm (2 sensor/cm^2^). Each sensor was calibrated using a standard calibration device (Trublu Calibration, Novel, Munich, Germany). The insole was connected to the Pedar-X box, which was attached to the waist of each participant. The insoles were inserted in the right (dominant leg) shoe of each participant during the data collection. The Pedar-X data acquisition software was used to collect and filter the data. Only the data on the right foot (landing foot) were collected, and the sample frequency was set to 200 Hz. The system has been demonstrated to be a valid and reliable plantar pressure measurement system [14].

The study was performed on a standard badminton court. The court laminated PVC flooring and wooden flooring. Prior to data collection, each participant was asked to perform a maximum lunge to measure individual maximum lunge distances at the right, left, and front directions. (Fig 1) The participants were then instructed to begin the test from the start positions, the marking of which was based on the measured distance for each participant. From the start position, each participant performed a maximum lunge, hit the target, and returned to the start position. If a player completely accomplished all three steps to hit the shuttlecock and recover to the starting position, then the lunge task was deemed successful. The distances of the three successful lunge step trials were measured and averaged to determine the landing position of each participant [6]. Prior to testing, the same tester who could consistently perform the respective badminton movements demonstrated the footwork and foot placements of the typical lunge steps at the three directions to all the participants. A badminton coach determined successful lunge task in testing. Each participant must performed five successful tasks with three shoe brands at the three directions. A total of 15 successful trials were collected for each shoe type. The participants were provided with 30 s and 5 min rests between trials and lunge directions, respectively, to minimize fatigue. Both the lunge directions and the shoe types were randomized across the participants. During testing, the shoes and different lunge tasks were randomly assigned to the participants.

Using the Novel Pedar-X system software, the plantar surface was initially divided into four larger areas: rearfoot (30% of foot length), midfoot (30% of foot length), and forefoot (25% of foot length) and toes (15% of foot length). The rearfoot, midfoot, forefoot and toes were subdivided, respectively, into M1 (medial heel, 50% of the rearfoot width), M2 (lateral heel, 50% of the rearfoot width), M3 (medial midfoot, 50% of the midfoot width), M4 (lateral midfoot, 50% of the midfoot width), M5 (medial forefoot, 30% of the forefoot width), M6 (central forefoot, 40% of the forefoot width), M7 (lateral forefoot, 30% of the forefoot width), M8 (great toe, 25% of the toes width), and M9 (lesser toes, 75% of the toes width). The mask was used to determine plantar pressure of running in previous study [12, 15]. The plantar load data on the right foot were extracted while the participants performed the lunge tasks at three different directions. The peak pressures (PP), maximum force (MF), and contact area (CA) at the total foot and at each foot mask were measured by the insole pressure system.

### Statistical analyses

All data are reported as mean ± standard deviation (SD). The data were tested for normal distribution using the Kolmogorov–Smirnov test, and homoscedasticity was verified using Levene’s test. Two-way ANOVA with repeated measures (directions × shoes) was used to determine the effects of the different directions (right, left, and front) and different shoes (L, M, and Y), as well as the interaction of these two variables, on the measurements. When ANOVA revealed significant direction and direction-by-shoe interaction effects, ANOVA with repeated measures was employed to compare the changes in the measures among the directions or shoes. Significance was set to alpha<0.05, and Bonferroni adjustment was used to correct multiple measurements.

## Results

The mean age of subjects was 23.8 y (SD = 3.3 y). Their mean body height and mass were 169.3 cm (SD = 4.5 cm) and 62.67 kg (SD = 8.1 kg) respectively.

The mean distances of the maximum forward lunge tasks were 2.15 m(SD = 0.30 m) for the right-forward lunge, 2.07 m (SD = 0.25 m) for the left-forward lunge, and 2.07 m (SD = 0.25 m) for the front-forward lunge. No significant difference was found in the distances of the three forward lunges, between participants.

The mean and standard deviations of the PP, MF, and CA are presented in Table 1. The ANOVA did not indicate a significant shoe effect for all the testing variables, including the PP, MF, and CA variables.

**Table 1:** Plantar loads at each region by different lunges and shoes.

Variables	Shoes	Directions	Total	M1	M2	M3	M4	M5	M6	M7	M8	M9
MF(%BW)	L	Left	198.50±47.50	68.19±14.10	86.70±18.49	11.46±6.01	22.47±7.18	21.19±8.14	17.40±7.59	16.64±6.53	17.75±6.73	19.38±7.82
		Front	202.57±48.09	66.30±10.94	84.44±15.51	11.82±10.83	23.92±7.56*	19.49±9.33	17.06±8.36	16.93±6.56	15.23±5.80* †	17.51±7.25
		Right	207.73±66.15	65.25±15.15	74.63±20.52	13.35±8.63	25.15±9.49	22.15±11.29	18.66±9.12	17.80±7.98	18.29±7.59	19.66±8.41
	M	Left	204.18±52.97	70.41±11.50	81.06±16.29	15.33±10.83	26.98±8.35	22.50±12.04	15.15±7.86	14.47±5.57	20.50±7.80	21.74±7.77
		Front	212.69±56.97	69.61±13.70	78.13±17.40	17.67±11.03	28.90±9.07*	21.15±12.80	15.36±8.34	16.35±7.13	17.52±8.38* †	20.14±8.63
		Right	210.81±59.19	72.76±13.21	70.87±16.41	17.49±11.08	27.73±9.34	22.46±11.25	15.71±8.48	15.52±5.83	19.88±8.40	21.50±8.14
	Y	Left	198.87±37.84	75.52±11.07	81.62±18.31	10.03±4.99	21.57±7.62	20.67±9.03	14.85±6.87	16.77±6.55	17.70±6.37	23.19±8.68
		Front	208.71±42.75	76.89±12.41	83.79±15.31	9.87±5.29	23.12±7.34*	19.60±9.97	15.09±7.51	17.98±7.10	16.05±5.56* †	22.27±9.07
		Right	200.01±41.92	76.47±12.24	80.08±15.08	8.90±5.04	22.07±7.20	19.42±10.39	15.46±7.64	18.01±7.10	16.08±5.18	23.08±8.66
PP(KPa)	L	Left	483.51±97.95	426.66±108.14	467.45±96.96	74.83±21.71	100.99±19.71	141.20±54.00	119.93±52.26	99.71±29.10	191.46±79.42	123.86±39.16
		Front	478.50±102.95	428.25±118.48	462.44±96.09	73.21±24.74	102.88±21.64	131.20±60.91	112.18±55.05	99.31±30.00	161.16±53.04* †	118.86±45.07
		Right	453.89±113.09	403.22±133.81	422.42±125.05	71.61±28.33	102.50±32.12	150.47±96.18	126.07±64.02	110.61±36.75	193.38±82.15	127.50±40.29
	M	Left	459.89±115.11	419.16±119.90	434.00±130.89	100.85±42.49	117.57±32.71	163.39±96.73	116.45±64.93	108.25±29.05	234.78±.91.65	152.93±47.47
		Front	463.73±125.53	417.48±131.68	438.98±150.84	105.60±42.77	120.42±35.80	154.48±93.17	111.00±59.96	113.76±29.31	205.80±94.27* †	148.10±62.84
		Right	471.35±125.98	447.32±133.51	448.38±137.67	102.06±44.36	116.23±36.20	157.17±77.95	113.52±56.22	117.50±30.52	224.22±84.91	151.94±53.86
	Y	Left	516.00±112.13	476.06±111.76	495.40±109.13	81.07±19.69	98.77±24.22	150.43±66.70	112.33±49.16	100.13±35.76	196.76±78.60	143.96±47.75
		Front	504.92±84.29	475.09±77.64	496.87±85.74	80.93±21.07	99.88±19.72	141.09±67.30	113.92±54.29	102.13±33.12	175.53±66.56* †	138.52±49.68
		Right	495.43±100.87	474.42±98.27	477.88±96.00	73.44±19.03	92.59±18.53	141.74±71.19	116.57±55.95	104.30±32.90	181.58±61.06	142.29±49.31
CA(cm^2^)	L	Left	151.96±14.60	22.71±0.00	22.25±0.00	15.84±6.40	22.25±3.60	14.10±2.17	14.80±1.91	14.53±2.80	8.93±0.89	15.31±3.64
		Front	150.40±17.29	22.42±1.14	22.25±0.00	15.53±7.17	22.55±3.32	13.93±2.20	14.18±2.90	14.52±2.28	8.64±1.10	15.01±3.39
		Right	152.85±15.31	22.34±1.12	21.73±2.00	16.56±7.18	22.93±2.02	14.86±1.69	14.61±2.63	14.29±2.60	8.86±0.93	15.41±3.24
	M	Left	149.63±17.05	22.41±1.14	21.89±1.37	15.99±5.51	22.93±1.85	13.69±2.03	13.27±3.76	13.62±3.93	8.84±0.85	15.78±3.34
		Front	149.63±18.32	22.39±1.14	21.89±1.37	17.09±5.79	23.18±1.58	13.29±2.39	13.42±3.92	13.85±3.69	8.20±1.55	15.13±3.58
		Right	151.69±16.79	22.41±1.14	21.96±1.11	17.21±6.00	23.12±1.56	14.09±2.16	13.58±3.76	13.92±3.17	8.67±0.96	15.13±3.58
	Y	Left	146.69±15.01	22.23±0.09	22.23±0.09	13.04±6.17	21.47±4.48	12.68±2.25	12.86±3.09	15.00±1.54	9.06±0.85	16.54±3.20
		Front	146.73±14.51	22.71±0.00	21.89±1.37	12.83±6.11	22.02±3.43	12.74±2.31	12.90±3.29	14.96±1.62	8.76±1.29	16.44±3.18
		Right	151.96±14.60	22.71±0.00	22.13±1.32	12.26±6.56	21.84±3.93	12.56±2.53	13.10±2.81	14.77±1.86	8.50±1.33	16.68±2.73

Note: Values are mean±SD

MF, maximum force; PP, peak pressure; CA, contact area; The three pairs of shoes were coded as L, M, and Y.

M1, medial heel, M2, lateral heel, M3, medial midfoot, M4, lateral midfoot, M5, first metatarsal head, M6, second and third metatarsal head, M7, fourth and fifth metatarsal heads, M8, great toe, M9, lesser toes

* p < 0.05 left lunge vs. front lunge

† p < 0.05 right lunge vs. front lunge

**Fig 2:**
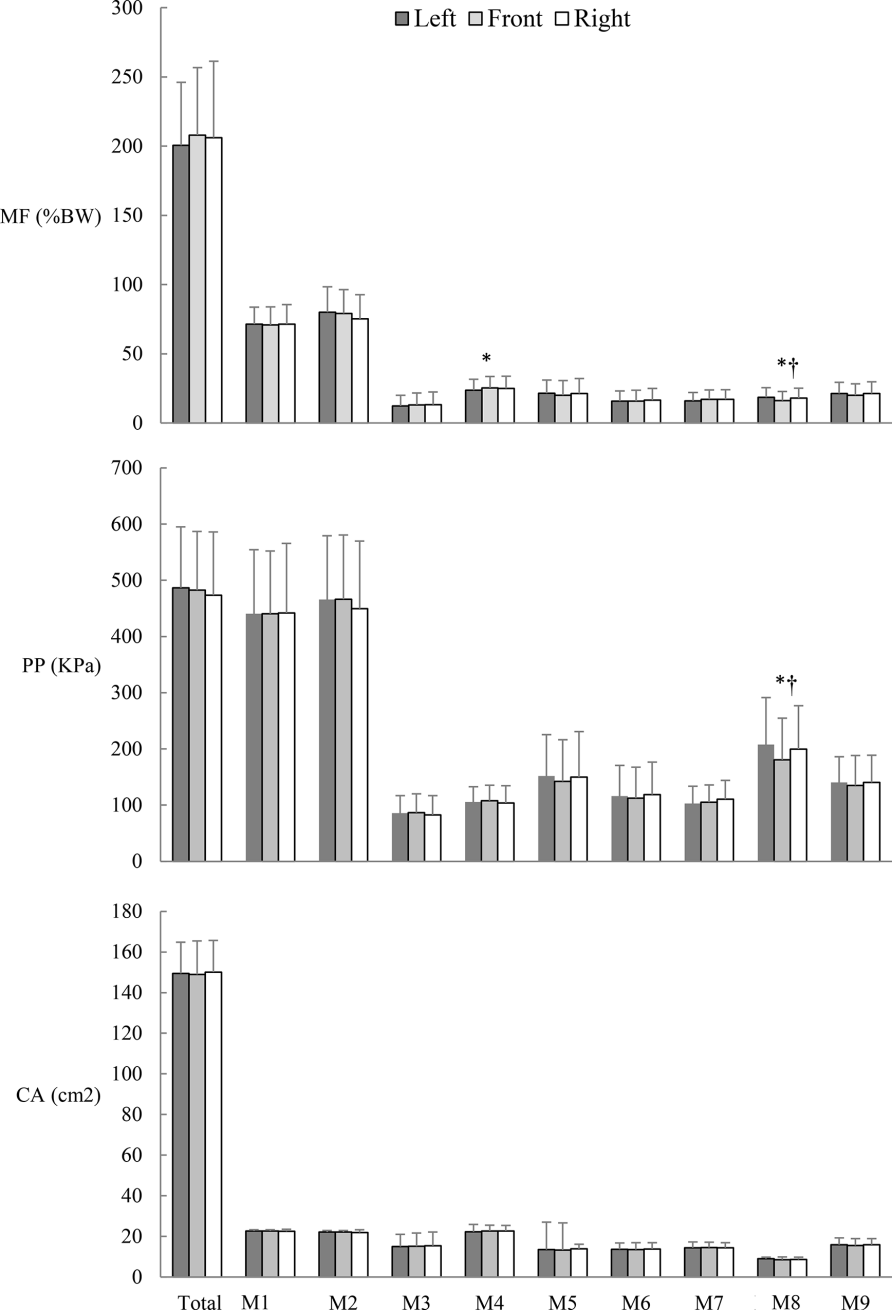
Comparison of insole loading parameters for maximum lunges at different directions. MF, maximum force; PP, peak pressure; CA, contact area; M1, medial heel, M2, lateral heel, M3, medial midfoot, M4, lateral midfoot, M5, first metatarsal head, M6, second and third metatarsal head, M7, fourth and fifth metatarsal heads, M8, great toe, M9, lesser toes. * *p* < 0.05 left lunge vs. front lunge. † *p* < 0.05 right lunge vs. front lunge.

As shown in Fig 2, the MF on the lateral midfoot was lower when performing the left-forward lunge than when performing the front-forward lunge (p = 0.006, 95% CI = −2.88 to −0.04%BW). Meanwhile, the MF on the great toe region was lower when performing the front-forward lunge than when performing the right-forward lunge (p = 0.047, 95% CI = −3.62 to −0.02%BW) and the left-forward lunge (p = 0.015, 95% CI = −4.39 to −0.38%BW). No significant differences were found in the MF on the other regions for the three lunges.

With regard to PP, no difference was observed in the PP on all regions for the three lunges, except for the PP on the great toe region. The PP on the great toe region was lower for the front-forward lunge than for the right-forward lunge (p = 0.048, 95% CI = −37.63 to −0.16 KPa) and the left-forward lunge (p- = 0.008, 95% CI = −47.76 to −5.91 KPa).

No significant difference was observed in the CA on all the regions for the three lunges. (Fig 2)

## Discussion

We investigated the effects of the three maximum forward lunges on in-shoe plantar loads. We also identified the most critical characteristics of plantar loads during one-step maximum forward lunges. Comparable plantar loads were observed among the three lunges and among the three shoe brands. The front-forward lunge showed lower plantar loads on the great toe region compared with the right- and left-forward lunges.

In general, badminton shoes may improve the performance of a player while preventing excessive load and relevant sports injuries through optimal shock attenuation and movement stabilization [4, 9]. The three shoe brands considered in the present study showed similar in-shoe plantar loads. This result is consistent with that reported by a recent study, which examined the kinetics of players during lunges while wearing two pairs of badminton shoes of different brands and found no significant shoe effect on all the ground reaction forces and in-shoe plantar loads [4]. These results may indicate that different brands of badminton shoes could provide similar shock attenuation for badminton players. Furthermore, sports surfaces are known to influence the load absorption and absorption mechanism. Previous studies have discussed plantar pressures when performing different sports tasks on different sports surfaces [12, 15]. Badminton players perform various movements on different sports surfaces. The sports surfaces may influence plantar pressure during badminton lunge tasks. Therefore, further studies are recommended to determine the plantar pressure during lunge tasks on different sports surfaces.

In badminton competitions, players often perform forward lunges to hit the shuttlecock. Thus, they are repeatedly burdened by vGRF, the peak of which is approx [4, 6, 9]. Lee et al. found that the mean peak vGRF is approximately 2.2 times the BW for right-forward lunges [9].The peak vGRF has also been found to be higher for the left-forward lunge (2.44 times the BW) than for the right-forward lunge (2.36 times the BW) [4]. In the current study, the average maximum force of the foot-and-shoe interaction was approximately 1.99 to 2.12 times the BW. Footwear material and structural properties can be optimized to attenuate external impact forces [16]. Therefore, the small impact force in the current study may be explained by the shock attenuation of the badminton shoes.

In the current study, no difference was observed in the plantar loads for the maximum left- and right-forward lunges. Consistent with our work, another study did not find a significant difference in plantar pressure for the one-step right- and left-forward lunges [8]. Hong et al. recently investigated the in-shoe peak plantar pressure during left- and right-forward lunges [4]. They found that the left-forward lunge has greater plantar pressure than the right-forward lunge for the total foot and heel regions. The discrepancy in the plantar pressure found in the current study and in the work of Hong et al. may be caused by different lunge settings. The present study employed one-step maximum forward lunges, whereas Hong et al. examined plantar pressure during continuous lunge steps. In the current present study, the MF and PP on the great toe region were higher for the left and right lunges than for the front lunge. This result indicates that the left and right maximum lunges may be the most critical movements for long-distance forward lunges. Furthermore, kinematic adjustments differ among different lunge movements [1].

Jorgensen and Winge reported that 58% of badminton injuries occur in the lower extremities while 42% occur in the upper extremities and the back. Of these injuries, 74% are injuries that are overused, 23% are various sprains, 1.5% are bone fracture and 1.5% are contusion [17]. In a badminton game, the frequent execution of a lunge step is generally considered as a major risk factor of injuries to the lower extremities [4]. Researchers suggest that the Achilles tendon, plantar fascia, anterior talo-fibular ligament of the specific musculotendon, and ligamentous structures are more susceptible to severe injury risks in badminton than in other sports because of the unique and repetitive movements, such as frequent stop-and-go maneuvers, required in a badminton game [18]. The feet experience a great amount of stress during lunges. In the current study, the MF and PP in for maximum forward lunges were identified, especially those on the heel regions. The heel experiences an impact force of more than 70% of the BW and plantar pressure of 440 KPa during maximum lunges. In our previous study, we investigated the in-shoe loads of running [12, 15]. In general, the heel only experiences an impact force of about 55% of the BW and plantar pressure of 250 KPa during a 3.8 m/s run on a concrete surface [12, 15]. Differences in in-shoe plantar loads on the heel region may exist between running and lunges. The difference in plantar loads may be caused by different kinetic and kinematic adjustments for different movements. Furthermore, the differences in plantar loads may influence various sports injuries.

Although the findings of the current study demonstrate differences in in-shoe plantar loads for different forward lunges, these findings are merely recommendations that were derived in terms of plantar load. A comprehensive evaluation of the biomechanics of the lower extremities in typical footwork forms for different sports is necessary to provide further insights into the biomechanics of sports injuries. Further research will provide useful information for establishing evidence-based improvements in athletic performance, prevention of sports injuries, and design of badminton footwear. The findings of the current study may have been impacted by limitations inherent to the overall study design. Only male right-handed players recruited in the cuurent study, the results may not suit female and/or left heanded badminton players. Play surfaces and shoes design were not considered in study design, this is another limitation in the current study.

In conclusion, the investigation into in-shoe plantar loads for maximum forward lunges indicated that compared with the front-forward lunge, left- and right-forward lunges induce higher plantar loads at great toe region on the dominant leg of male right-handed badminton players. Thus, differences in the plantar loads for different lunge directions may be potential risks for overuse-related injuries to the lower extremities of badminton players.
